# Stochastic Hypothesis of Transition from Inborn Neutropenia to AML: Interactions of Cell Population Dynamics and Population Genetics

**DOI:** 10.3389/fonc.2013.00089

**Published:** 2013-04-29

**Authors:** Marek Kimmel, Seth Corey

**Affiliations:** ^1^Department of Statistics, Rice UniversityHouston, TX, USA; ^2^Department of Bioengineering, Rice UniversityHouston, TX, USA; ^3^Department of Pediatrics, Feinberg School of Medicine, Northwestern UniversityChicago, IL, USA; ^4^Department Cell and Molecular Biology, Feinberg School of Medicine, Northwestern UniversityChicago, IL, USA

**Keywords:** severe congenital neutropenia, myelodysplastic syndrome, acute myeloid leukemia, branching process, driver mutations, clonal evolution

## Abstract

We present a stochastic model of driver mutations in the transition from severe congenital neutropenia to myelodysplastic syndrome to acute myeloid leukemia (AML). The model has the form of a multitype branching process. We derive equations for the distributions of the times to consecutive driver mutations and set up simulations involving a range of hypotheses regarding acceleration of the mutation rates in successive mutant clones. Our model reproduces the clinical distribution of times at diagnosis of secondary AML. Surprisingly, within the framework of our assumptions, stochasticity of the mutation process is incapable of explaining the spread of times at diagnosis of AML in this case; it is necessary to additionally assume a wide spread of proliferative parameters among disease cases. This finding is unexpected but generally consistent with the wide heterogeneity of characteristics of human cancers.

## Introduction

Granulocytes are essential for host defense and survival. Their importance is apparent in severe congenital neutropenia (SCN). Life-threatening infections in children with SCN can be avoided through the use of recombinant granulocyte colony-stimulating factor (GCSF). However, SCN often transforms into secondary myelodysplastic syndrome (sMDS) and then into secondary acute myeloid leukemia (sAML). A great unresolved clinical question is whether chronic, pharmacological doses of GCSF contribute to this transformation (Glaubach and Corey, 2012). A number of epidemiological clinical trials have demonstrated a strong association between exposure to GCSF and sMDS/sAML (Dong et al., 1995; Donadieu et al., 2005; Rosenberg et al., 2006; Germeshausen et al., 2007; Carlsson et al., 2012). Mutations in the distal domain of the GCSF Receptor (GCSFR) have been isolated from patients with SCN who developed sMDS/sAML or patients with *de novo* MDS (Beekman and Touw, 2010). Most recently, clonal evolution over approximately 20 years was documented in a patient with SCN who developed sMDS/sAML (Beekman et al., 2012). Clonal evolution of a sick hematopoietic progenitor cell in SCN involves perturbations in proximal and distal signaling networks triggered by a mutant GCSFR. Transition from SCN → sMDS → sAML involves chance mechanisms such as mutations, drift and transcription, and receptor noise, which require that stochastic models are needed (Whichard et al., 2010).

In the present paper we use stochastic modeling to understand the wide range of times at which the transition to sAML occurs. We develop a model in the form of a multitype branching process, which allows one tying population genetics and population dynamics aspects of the transition from SCN to sMDS to sAML, and validating it against existing evidence. Branching processes have been used widely to model mutation, selection, and drift processes in populations of variable size, to which the classical Wright–Fisher model does not apply (Cyran and Kimmel, 2010). We adopted approach similar to that developed in Nowak’s group (Bozic et al., 2010), modified to bring out stochastic time intervals between successive driver mutations.

The model we developed allows predicting the time at transition to sAML given the probability of each successive driver mutation, the number of mutations needed, and the proliferative potential of each successive mutated clone of hemopoietic stem cells. We can then compare these times to observed distribution of times at transition. As documented in the paper, the outcome is intriguing: stochasticity inherent in the mutation process is insufficient to explain the wide distribution of times at transition (ranging from 1 to 38; Table 1). Additional factors are required, one of which may be a wide interpatient spread of proliferative potential of the mutant clones.

**Table 1:** **Summary of life histories of patients transitioning from severe congenital neutropenia (SCN) to secondary myelodysplastic syndrome (sMDS) to secondary acute myeloid leukemia (sAML) (Walter et al., 2012)**.

Phase of disease	Age at diagnosis (years)	Number of co-existing mutations
SCN	0–0.5	1*
MDS	1–12	1–3 ± chromosomal loss or gain**
AML	2–38	1–9 ± chromosomal loss or gain***

*ELANE, HAX1, G6PC, WAS, CSF3R.

**GCSF3R, ZC3H18, LLGL2; RAS ± monosomy 7.

***RUNX1, ASXL1, p300, CEBA, CSF3R, MGA,SUZ12, LAMB,FBXO18,CCDC15, ± monosomy 7, trisomy 21.

### Population genetics and population dynamics model of the SCN → sMDS → sAML transition

Missense, nonsense, and frameshift mutations, and dysregulated alternative splicing in GCSFR have been isolated in patients with MDS/AML. In the study of Beekman et al. (2012), nonsense and missense mutations in GCSFR arose during the course of the disease. In the model we envision, population genetics, and population dynamics of proliferating bone marrow cells are closely intertwined.

### Population genetics perspective

**Figure 1:**
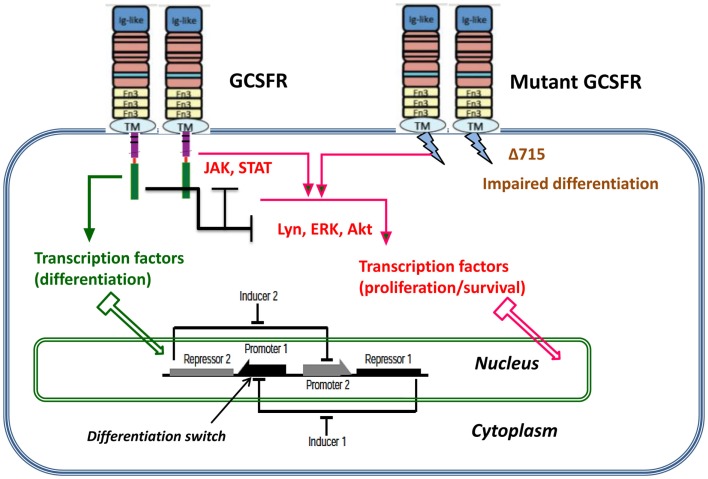
**Dynamic stochastic model of impaired differentiation in granulocyte precursors**. GCSF signaling occurs through its cognate receptor, GCSFR. It involves both proximal signaling networks consisting of signaling molecules such as Lyn, Jak, STAT, Akt, and ERK, and distal gene regulatory networks consisting of transcription factors. Together, these signaling networks promote proliferation, survival, and differentiation. In patients with severe congenital neutropenia, the earliest known mutation to contribute to transformation to secondary MDS or AML is a nonsense mutation in the GCSFR gene. This mutation leads to a truncated receptor, GCSFR delta 715.

Proliferating healthy cells in the bone marrow mutate at random times, possibly influenced by super-pharmacological doses of GCSF. A summary of possible mutations and their consequences for proliferation dynamics of granulocyte precursors is depicted in Figure 1. GCSF signaling occurs through its cognate receptor, GCSFR. It involves both proximal signaling networks consisting of signaling molecules such as Lyn, Jak, STAT, Akt, and ERK, and distal gene regulatory networks consisting of transcription factors. Together, these signaling networks promote proliferation, survival, and differentiation. In patients with SCN, the earliest known mutation to contribute to transformation to secondary MDS or AML is a nonsense mutation in the GCSFR gene. This mutation leads to a truncated receptor, GCSR delta 715 (Glaubach and Corey, 2012, and reference therein).

It follows from a simple calculus of mutation events that as long as the cell population size is kept in check, the rate at which new mutant clones appear in the population is rather low. When the population expands, new mutant clones arise faster (see further on).

In our model we take the view that carcinogenesis is driven by a succession of small-scale (e.g., point) mutations in specific loci. Other viewpoints (epigenetic effects, karyotypic alterations, intercellular interactions, etc.) have been suggested. In treatment-related MDS some drugs (e.g., many alkylating agents) induce t-MDS primarily via large scale alterations that lead to karyotypic instability (Bhatia, 2011).

### Population dynamics perspective

Limited mutation load at the SCN phase causes neutropenia and fluctuations of cell population size. With time, accumulation of driver mutations causes expansion of mutant clones, which however are not yet expanding at a dramatic rate. At some point in time, mutations accumulate sufficiently to cause a major change in the proliferation law and the now malignant cell population starts rapidly expanding.

**Figure 2:**
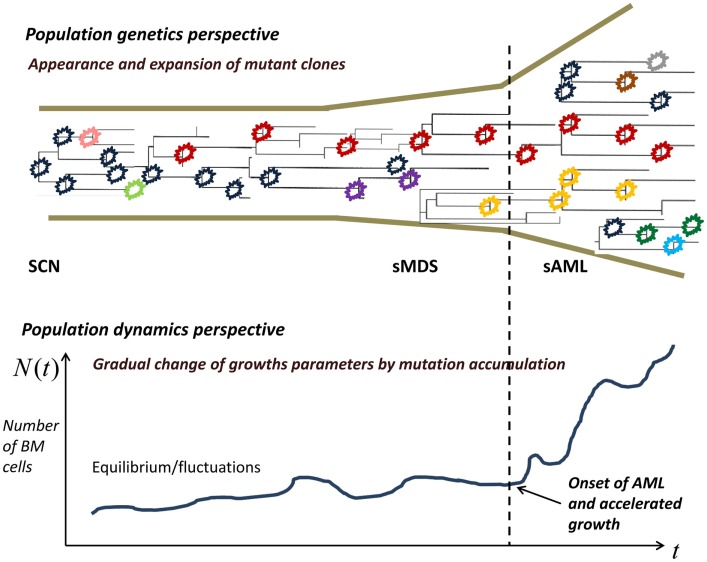
**Proliferating healthy cells in the bone marrow mutate at random times, possibly influenced by super-pharmacological doses of GCSF**. As long as the cell population size is kept in check, genetic drift, and selection remove many of the mutants, whereas some mutants persist. When the population expands, new mutant clones become more easily established. At some point, a qualitative change in the proliferation rate occurs and the now malignant cell population starts rapidly expanding.

Our model is based on the following hypotheses (Figure 2):

1. At the time of diagnosis of SCN, GCSF therapy is initiated, which induces an initial series of *X* driver mutations, occurring at random times.
2. The *X*-th mutation causes transition to the MDS, during which further *Y* mutations occur.
3. After *X* + *Y* mutations, the AML stage begins, during which the subsequent mutant clone grow at increasing rate, which in turn shortens times at which still new mutations appear.

In the model, the increasing proliferation rate of successive mutant clones causes acceleration of growth of the malignant bone marrow stem cell population, which shortens the time interval to appearance of new clones, which in turns increases proliferation rate, and so forth; this results in a positive feedback. As we will see, the stochastic nature of the process (the times to appearance of each next mutant are random) causes a spread of the timing of the subsequence mutations, particularly the first *X* mutations during the SCN phase. This may result in the transition to MDS not manifesting itself for a very long time in a fraction of cases.

### Role of stochastic dynamics in the model

We explain some other intuitions underlying the model. For a new subclone, stochastic theory is used to estimate extinction probability, with extinction after more than a few cell generations being negligible in view of the growth advantage of the new clone. However the time at which the next mutation occurs in a cell clone is also stochastic and it is as a rule more dispersed for the slower-growing clones. Therefore the time to reaching the threshold number of bone marrow stem cells (which in our model defines the time at sAML diagnosis), is a random variable. One of the questions we ask is if dispersion of this time matches the wide distribution of the times at diagnosis (Rosenberg et al., 2010).

## Materials and Methods

### Mathematics of the model

The population-genetic effect of population size-dependent accumulation of mutations occurs as a natural consequence of the proliferation law in the form of a multitype Galton–Watson branching process:

1. Consecutively arising surviving mutant clones are numbered with the index *k*, ranging from 1 to *K*; time interval between the appearance of the *k*-th and *k* + 1-st surviving mutant clones is denoted by τ*_k_*. *k*-th mutant cells have accumulated *k* driver mutations (assuming the clone in SCN bone marrow at diagnosis has a single cell with one driver mutation, which seems a defendable idealization).
2. All clones expand as Galton–Watson branching processes (see further on). Cell life length is constant and equal to *T*, and at that time the cell either produces two progeny with probability *b_k_* (cell type *k*) or dies (or becomes quiescent or differentiated, which does not make a difference for disease dynamics) with probability 1 − *b_k_*.
3. A cell of type *k* can mutate upon its birth (for definiteness) to type *k* + 1 with probability *u*.

These three rules allow one derive the probability distributions of time intervals τ*_k_*, probabilities of survival of each clone, and expected growth laws of each clone. Mathematical details follow from the theory of Galton–Watson branching process; see for example the monograph by Kimmel and Axelrod (2002).

We assume that cell division is effective with probability *b*, i.e., the probability generating function (pgf) of the number of progeny cells per parent cell has the form *f*(s) = *bs*^2^ + (1 − *b*). The extinction probability *q* is the smaller solution of the equation *q* = *f*(*q*), which is less than 1 if the process is supercritical. In our case,

(1) $$q=bq^{2}+\left(1-b\right)\Rightarrow q=\left(b^{-1}-1\right);\;b\in \left(0.5,\;1\right].$$

Similarly, the expected number of progeny of a cell is equal to *f* ′(1−) = 2*b*, hence the expected number of cells at time *t* is equal to *N*(*t*) = (2*b*)^(*t*/*T*)^, which yields the value of λ

(2) $$\exp\left(\lambda t\right)={\left(2b\right)}^{\left(t∕T\right)}\Rightarrow \lambda =\ln\left(2b\right)∕T.$$

We will use “continuous” time *t* for notational convenience. However, we consider generations of cells dividing at discrete times *t_i_* = *iT*, where *T* is the average cell cycle time. As it is known, the expected (mean) growth law in the Galton–Watson process has the form

(3) $$\begin{array}{rcll} & \text{E}\left[\text{\# cells, at time }t,\text{ in a clone started at time }t_{0}\right]\;\overset{def}{=} & & \\ & \;\;\left[N\left(t\right)=\exp\left(\lambda \left(t-t_{0}\right)\right)\right],\text{ as }t\to \infty . & \end{array}$$

To determine the distribution of time to a mutation creating a new non-extinct clone, we consider a newborn cell. In this cell, mutation may occur with probability *u*, and if the extinction probability of the mutant clone is *q*′, then the probability that the cell does not produce a new mutant clone is equal to 1 − *u*(1 − *q*′). Until time *t_i_* = *iT*, approximately $\overset{i}{\underset{j=0}{\sum }}N\left(t_{j}\right)=N\left(t_{0}\right)\left(\exp{\left(\lambda T\right)}^{i+1}-1\right){\left(\exp\left(\lambda T\right)-1\right)}^{-1}$ new cells are born, and assuming independence, we obtain

(4) $$\begin{array}{c} \mathrm{Pr}\;\left[\text{no mutant initiating nonextinct clone appears until}\;\text{time }t_{i}=iT\right]\\ =\mathrm{Pr}\;\left[\tau =:\text{time to nearest nonextinct}\;\text{mutant clone }>\;t_{i}=iT\right]={\left(1-u\left(1-q^{\prime }\right)\right)}^{\overset{i}{\underset{j=0}{\sum }}N\left(t_{j}\right)}\\ \;={\left(1-u\left(1-q^{\prime }\right)\right)}^{N\left(t_{0}\right)\frac{\exp{\left(\lambda T\right)}^{i+1}-1}{\exp\left(\lambda T\right)-1}}=a^{d\left(\exp\left(ct\right)-1\right)}. \end{array}$$

where, for the *k*-th mutant population

$$\begin{array}{cc} a & =\left(1-\left(1-q_{k+1}\right)u_{k}\right)=\left(1-u_{k}\left(2b_{k+1}-1\right)∕b_{k+1}\right),\\ c & =\lambda_{k}=\ln\left(2b_{k}\right)∕T,\\ d & ={\left(\exp\left(\lambda_{k}T\right)-1\right)}^{-1}={\left(2b_{k}-1\right)}^{-1}. \end{array}$$

Since the distribution tail of random variable τ*_k_* has the form

$$\mathrm{Pr}\left[\tau_{k}>\tau \right]=a^{d\left(\exp\left(ct\right)-1\right)}=\exp\left(-\ln\left(a^{-1}\right)d\left(\exp\left(c\tau \right)-1\right)\right).$$

it can be algorithmically generated using the inverse tail method

(5) $$\tau_{k}=c^{-1}\ln\left(\ln r∕\left(d\ln a\right)+1\right),$$

where *r* is a pseudo-random number uniformly distributed from 0 to 1. In this framework, a sample path of the number of cells in the *k*-th mutant clone (which contains cells with *k* mutations accumulated) is equal to

(6) $$N_{k}\left(t\right)=\left\{\begin{array}{cc} 0\; & t\leq \sum_{j=1}^{k-1}\tau_{j}\\ \exp\left(\lambda_{k}\left(t-\sum_{j=1}^{k-1}\tau_{j}\right)\right)\; & \text{otherwise} \end{array}\right.$$

The derivations presented are quite similar to those of Bozic et al. (2010), except that in that paper, expected times *E*(τ*_k_*) to the next mutation have been used. Here, we are interested in exposing stochastic variability in the time course of the SNC → sMDS → sAML transition. Another refinement would be to use distributions of cell counts instead of expected values *N_k_*(*t*). This would result in serious computational problems, arguably without much impact on the results.

### Modeling the SNC → sMDS → sAML transition

Equations 1 and 2 allow generating realizations of times to successive driver mutations under different values of mutation rates and proliferative characteristics of the mutant clones. We make the following assumptions:

1. Transition to sMDS requires one or two somatic driver mutations, whereas the transition to sAML requires at least three somatic driver mutations (cf. Table 1).
2. Diagnosis of sAML requires presence of 10^4^ leukemic HSC. For details of computations leading to this estimate, see further on.
3. Successive mutant clones have increasing proliferative potential. We assume a power law for the coefficients *b_i_*, which seems to lead to fits that do not contradict data:
  (7) $$b_{i}=\min\left(0.5+A\left(\varepsilon +{\left(i-1\right)}^{\kappa }\right),\;\;1\right),$$
  where coefficients *A*, ε, and κ are considered further on.
4. As it will be seen, it is necessary to assume that the coefficients *A* be generated from a probability distribution instead of assuming a constant value. We assume the distribution function *F_A_*(*a*) selected so that the times of at diagnosis of sAML fit available statistics (for details see further on).

### Estimate of the number of leukemic cells

We carried out computations based on two literature sources and then used rounding to the nearest order of magnitude to obtain a working threshold number of the leukemic initiating cells (LIC) (Bonnet and Dick, 1997). In both cases we assume that the volume of human bone marrow is equal to *V* = 1700 ml and that LIC cells constitute a fraction ψ = 10^−6^ of leukemic bone marrow mono-nucleated cells (BMMNC). We also assume that in sAML, fraction ρ = 0.8 of BMNNC is constituted by leukemic cells.

#### Estimate 1

Dedeepiya et al. (2012) provide an estimate of the number of BNNMC per 1 ml *B* = 3.67 × 10^6^. This results in an estimate of the number *L* of LIC cells in the entire bone marrow *L* = ρ × ψ × *V* × *B* = 4991 cells.

#### Estimate 2

Bender et al. (1994) provide estimates of *B* in the range from 3.02 × 10^6^ to 4.71 × 10^6^. This results in *L* = 4107 ÷ 5535 cells. These estimates are remarkably consistent. Rounding to the nearest order of magnitude results in a working estimate of *L* = 10^4^ cells.

### Time at diagnosis of sAML and distribution of parameter A

Under given values of parameters κ and ε as well as mutation rates *u_k_*, the time at diagnosis of sAML, defined as the time *T* from initiation of GCSF treatment such that

$$\underset{k}{\sum }N_{k}\left(T\right)=L$$

depends on parameter *A* according to an approximate power law

$$T=f\left(A\right)=\exp\left(\alpha \right)A^{\beta },$$

where β < 0. This dependence, which was obtained via simulation studies (not shown), allows finding the distribution of *A* that leads to a clinically observed distribution of the time of sAML diagnosis according to the following expression for distribution tails

$${\bar{F}}_{A}\left(a\right)=1-{\bar{F}}_{T}\left(\;f\left(a\right)\right),$$

where ${\bar{F}}_{T}\left(t\right)=\mathrm{Pr}\left[T>t\right]$ is the tail of the distribution of time *T*. This in turn allows generating pseudo-random realizations of *A* according to the expression

(8) $$A=f^{-1}\left({{\bar{F}}_{T}}^{-1}\left(R\right)\right)={\left(\exp\left(-\alpha \right){{\bar{F}}_{T}}^{-1}\left(R\right)\right)}^{1∕\beta },$$

where *R* is a pseudo-random number from the uniform distribution on the (0, 1) interval.

We need to approximate the tail of the distribution of the time at diagnosis of sAML. A recent source is the paper by Rosenberg et al. (2010). These authors reported results of a prospective study of 374 SCN patients, and included estimates of hazard rates and cumulative probability of sMDS/sAML as a function of time after GCSF treatment. Hazard rate grows for the first 5 years and then plateaus. To simplify computations we adopted a piecewise constant estimate of the hazard rate *h_T_*(*t*)

$$h_{T}\left(t\right)=\left\{\begin{array}{cc} 0.01\; & t\in \left[0,\;3\right)\\ 0.02\; & t\in \left[3,\infty \right) \end{array}\right.$$

with time in years. Comparing with Figure 1A in Rosenberg et al. (2010) we see that *h_T_*(*t*) remains within the confidence band computed based on the prospective study. Using the expression

$${\bar{F}}_{T}\left(t\right)=\exp\left(-\int_{\;0}^{\;t}h_{T}\left(\tau \right)d\tau \right)$$

and inverting the tail function ${\bar{F}}_{T}\left(t\right)$ we complete the derivation of expression Eq. 8 (elementary details not shown).

### Overview of parameter estimation

The form of expression Eq. 8 and plausible estimates of parameters κ and ε as well as of mutation rates *u_k_*, are difficult to be uniquely determined with the data available at the present time. We used the following heuristic procedure:

1. Driver mutation rates increase from the reference value by a factor of 5, starting mutation 3, so that *u*_1,2_ = *u* but *u*_3,4,5_ = 5*u*. The increase is needed for the later mutations to occur in quick succession, so that mutation 3 occurs before $\underset{k}{\sum }N_{k}\left(T\right)>L$, with *L* = 10^4^ being a relatively low value.
2. Reference driver mutation rate had to be set equal to 0.00034, 10 times higher than the value estimated by Bozic et al. (2010). This is required for enough mutations to accumulate before the threshold time *T*.
3. Proliferation rate increases as power κ of the mutation number, value κ = 2 provides sufficient acceleration to explain relative rapidity of the AML stage. The offset parameter ε = 0.02 keeps proliferation rate before mutation 1 sufficiently low.
4. Once estimates of parameters *u_k_*, κ, and ε are obtained, estimates of the power law parameters α and β are determined by a simulation study, and the generator of random parameter *A* is obtained via expression Eq. 8.

## Results

### Simulated course of disease

**Figure 3:**
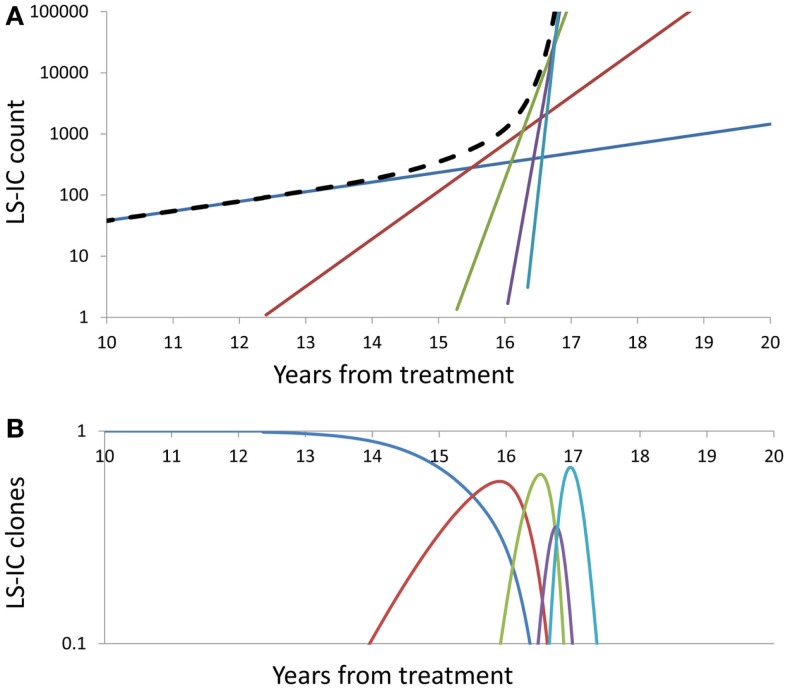
**Summary of successive driver mutations in the natural course of the SCN → sMDS → sAML transition**. **(A)** Counts *N_i_*(*t*) of cells in successive mutant clones, under model as in Eq. 7 with *A* = 0.02, ε = 0.2, and *k* = 2. Straight lines with increasing slopes: counts of cells in successive mutant clones. Thick dashed line: Total mutant cell count. **(B)** Relative proportions *n_i_*(*t*) = *N_i_*(*t*)/Σ*_j_N_j_*(*t*) of cells belonging to successive mutant clones. Further details as in the Section “Mathematics of the Model.”

Figure 3 depicts the impact of successive driver mutations on the natural course of the SCN → sMDS → sAML transition. Figure 3A depicts counts *N_i_*(*t*) of cells in successive mutant clones as a function of time, under model as in Eq. 7 with *A* = 0.005, ε = 0.02, and κ = 2. Straight lines with increasing slopes are counts of cells in successive mutant clones. We observe that the time intervals separating the origins of successive clones are decreasing with each mutation event. Thick dashed line represents the total mutant cell count. It is also interesting to observe that clones with increasing numbers of mutations dominate transiently, until they are replaced by other clones with higher proliferative capacity (selective value). Figure 3B depicts relative proportions *n_i_*(*t*) = *N_i_*(*t*)/Σ*_j_N_j_*(*t*) of cells belonging to successive mutant clones.

### Time at sAML diagnosis

**Figure 4:**
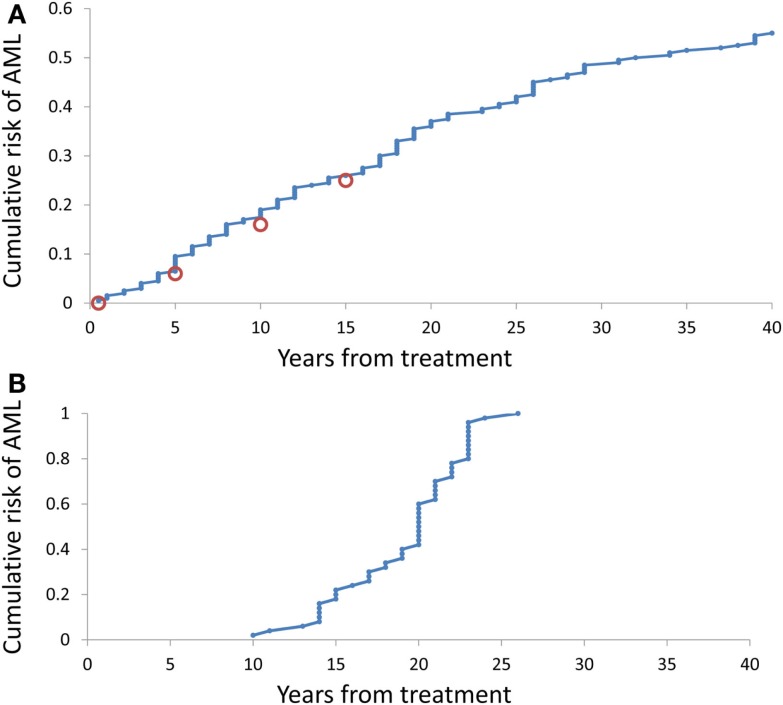
**Cumulative distributions of the model-generated times at diagnosis of sAML**. **(A)** Simulations under model as in Eq. 7 with *A* generated using Eq. 8, ε = 0.2, and *k* = 2. **(B)** Simulations under model as in Eq. 8 with *A* = 0.02, ε = 0.2, and *k* = 2.

It is somewhat surprising that under any combination of coefficients *A* and *k*, the range of simulated times at sAML diagnosis is rather narrow. Figure 4B depicts ranked simulated times at sAML diagnosis under model as in Eq. 7 with *A* = 0.005, ε = 0.02, and κ = 2. Spread of these values is narrow, with interquartile range between 15 and 21. Systematic simulation experiments demonstrate that this is the case for a wide range of *A* and κ parameter values. This outcome is in contrast to the wide spread of times at diagnosis summarized in Table 1 and that based on Rosenberg et al. (2010).

Simulation-estimation experiment outlined in the Methods demonstrates that distribution of simulated times (counting form initiation of CGSF treatment) at sAML (Rosenberg et al., 2010) is reproduced by our model. Figure 4A cumulative distribution of the times at sAML diagnosis under model as in Eq. 7 with κ = 2, ε = 0.02, and *A* generated from the distribution in Eq. 8 with α = − 0.655 and β = −0.912.

## Conclusion

The process of development and replacement of leukemic clones is influenced by the processes of genetic drift and selection (Walter et al., 2012). These forces are usually analyzed by geneticists in the framework of the Wright–Fisher or coalescent model (see Discussion and references in Cyran and Kimmel, 2010). However, in the case of expanding cell clones, the more appropriate population process seems to be one of the types of branching processes; in our case, the Galton–Watson process (Kimmel and Axelrod, 2002). In the particular version of the multitype Galton–Watson process that we use, genetic drift’s mechanism is the loss of variants through extinction and selection is embodied in the principle that each next surviving clone is proliferating faster (has greater fitness).

A characteristic feature of human cancers is very wide heterogeneity with respect to extent of involvement, genotype and rate of progression, and spread (Michor et al., 2004; Hanahan and Weinberg, 2011). This is in contrast to induced animal tumors, which are relatively uniform. Secondary AML, resulting from a transition from SCN via myelodysplastic syndrome, is not an exception, with onset varying from 1 to 38 years of age and with wide variability of mutational background (Table 1). It is interesting, and we consider it a major result, that such spread of the age of onset is not due solely to stochastic nature of mutation-driven transitions, but it requires a large variability in proliferative potential from one disease case to another. Also, this distribution of coefficient *A*, which parameterizes the proliferative potential, is right-skewed, with slowly evolving (low-*A*) clones prevailing. This provides a testable hypothesis about distribution of proliferating rates in leukemic stem cell clones.

The model presented in this paper addresses certain aspects of the SNC → sMDS → sAML transition. Among other, although we might derive an expression relating the number of driver (selective) mutations to the corresponding count of accumulated passenger (neutral) mutations (similarly as it was done in Bozic et al. (2010), we do not have at our disposal sequencing data to validate such an expression. Also, we do not attempt here to fit the distribution of the age at diagnosis of the sMDS, since we are missing data on individual life histories, which would involve somatic mutation as well as sequencing data.

From the mathematical point of view, the current model is also somewhat simplified. It considers each new mutation to provide more selective advantage to the arising clone. This is in apparent disagreement with the recent observation of Beekman et al. (2012), of mutations which appear at the sMDS stage and disappear at the sAML stage. The linear structure of mutation confers desirable simplicity to modeling but is not necessarily realistic. In the framework of multitype branching processes and special processes such as Griffiths and Pakes branching infinite allele model (Griffiths and Pakes, 1988; Kimmel and Mathaes, 2010), more complicated scenarios can be gaged.

## Conflict of Interest Statement

The authors declare that the research was conducted in the absence of any commercial or financial relationships that could be construed as a potential conflict of interest.
